# Announcement

**Published:** 2015-03-06

## National Kidney Month — March 2015

March is designated National Kidney Month to raise awareness about the prevention and early detection of kidney disease. In 2012, kidney diseases were the ninth leading cause of death in the United States (1). More than 10% (more than 20 million) of U.S. adults aged ≥20 years have chronic kidney disease (CKD), and most of them are unaware of their condition (2,3). Major risk factors for CKD include aging, diabetes, and high blood pressure. If left untreated, CKD can lead to kidney failure, requiring dialysis or transplantation for survival. However, controlling diabetes and high blood pressure can prevent or delay CKD and improve health outcomes (2).

In collaboration with partner agencies and organizations, CDC supports and maintains the CKD Surveillance Project website (http://nccd.cdc.gov/CKD/default.aspx) to document and monitor over time the burden of CKD in the United States, and to track progress in achieving *Healthy People 2020* objectives to prevent, detect, and manage CKD (4). CDC and its partners developed and disseminated the *National Chronic Kidney Disease Fact Sheet, 2014*, a consensus document about the burden of CKD in the United States that includes data on prevalence by race/ethnicity, risk factors, and health consequences (2). The National Kidney Disease Education Program developed *Making Sense of CKD: A Concise Guide for Managing Chronic Kidney Disease in the Primary Care Setting* (5) to help primary care providers identify, manage, and educate adult CKD patients. Information about kidney disease prevention and control is available at http://www.nkdep.nih.gov. Information about CDC’s CKD Initiative is available at http://www.cdc.gov/ckd.
